# CHML regulates migration and invasion in hepatocellular carcinoma via transcriptional and metabolic reprogramming

**DOI:** 10.3389/fonc.2025.1575809

**Published:** 2025-08-06

**Authors:** Huanqian Cao, Siyu Wang, Li Zhang, Heying Xie, Yiqiong Liu, Ruijiao Kong, Yin Jia, Ling Lu, Junfeng Jiang, Shanrong Liu

**Affiliations:** ^1^ Department of Laboratory & Diagnosis, Changhai Hospital, Navy Medical University, Shanghai, China; ^2^ Department of Pathology, Faculty of Medical Imaging, Naval Medical University, Shanghai, China; ^3^ Department of Pathogen Biology, Naval Medical University, Shanghai, China; ^4^ Histology and Embryology Department, Naval Medical University, Shanghai, China; ^5^ School of Gongli Hospital Medical Technology, University of Shanghai for Science and Technology, Shanghai, China

**Keywords:** gene editing, CHML, hepatocellular carcinoma, neoplasm metastasis, choline, metabolism

## Abstract

**Background:**

Hepatocellular carcinoma (HCC), a prevalent malignant neoplasm, presents significant therapeutic challenges. However, the key factors and mechanisms driving HCC metastasis remain incompletely understood. This study aimed to elucidate the mechanism through which CHML regulates the migration and invasion of HCC cells.

**Methods:**

Following CHML knockout or overexpression, we assessed the proliferative capacity of HCC cells using the Cell Counting Kit-8 (CCK-8) assay, 5-ethynyl-2′-deoxyuridine (EdU) incorporation, colony formation assay, and subcutaneous xenograft tumor models in nude mice. Cell migration and invasion were evaluated using wound healing and Transwell assays. We utilized transcriptome sequencing and untargeted metabolomics to assess the gene's effects on transcriptomic and metabolic changes and its mechanisms in regulating migration.

**Results:**

CHML knockout significantly inhibited the migration and invasion of HCC cells in vitro, whereas CHML overexpression promoted these phenotypes (*P*<0.05). Transcriptomic sequencing revealed CHML-mediated regulation of migration-associated pathways, whereas untargeted metabolomics identified choline metabolism as a key significantly altered pathway. Notably, the integration of transcriptomics and untargeted metabolomics identified choline metabolism as a pivotal pathway in CHML-regulated migration and invasion. The subsequent mechanistic analysis demonstrated that CHML upregulated the Solute carrier family 44 member 3 (SLC44A3) to enhance choline uptake, thereby increasing phosphatidic acid (PA) production. This metabolic shift activated MAPK and PI3K-AKT signaling cascades, ultimately driving HCC cell migration and invasion.

**Conclusion:**

CHML promoted the migration and invasion of HCC cells through multiple pathways. Our findings provide novel insights into metabolic dependencies in HCC metastasis and position CHML as a promising therapeutic target.

## 1 Introduction

Hepatocellular carcinoma (HCC) represents the most common primary liver malignancy and ranks as the fourth leading cause of global cancer-related mortality, accounting for approximately 830,000 deaths annually (1). While hepatic resection and radiofrequency ablation offer effective treatment for early-to-intermediate HCC, most patients present with advanced-stage disease, precluding surgical intervention. Advanced HCC exhibits high metastatic potential, contributing to poor prognosis and constituting a primary cause of treatment failure and reduced survival (2). Metastasis is also one of the main reasons for treatment failure and low survival rates in the majority of HCC patients. Existing molecular targeted agents (e.g., sorafenib) provide limited survival benefits for advanced HCC but fail to prevent recurrence or metastasis (3, 4). Thus, elucidating the molecular mechanisms underlying HCC metastasis is critical for developing novel therapeutic strategies.

CHML(Rab escort protein-2; REP-2) facilitates Rab protein prenylation, a process essential for Rab membrane localization and function. Its homolog CHM/REP-1 promotes cervical, lung, and colorectal cancer progression (5), and its mutation can lead to human choroideremia, ultimately causing choroidal neovascularization (6). CHML overexpression correlates with poor prognosis in lung adenocarcinoma (7) and multiple myeloma (8). *In vitro* experiments reported by Dong et al. revealed that miR-199a-3p targets CHML, which can promote the growth of non-small cell lung cancer cells by binding to Rab5A (9). In HCC, our prior collaborative work demonstrated that CHML escorts Rab14 to the membrane, sustaining Rab14 recycling to enhance tumor cell migration and invasion (10). However, whether CHML contributes to HCC progression through additional mechanisms, particularly via global transcriptional or metabolic reprogramming, remains unknown. Furthermore, the consequences of CHML ablation on HCC proliferation, migration, and invasion require definitive validation.

In this study, we successfully knocked out the CHML gene in liver cancer cells using CRISPR-Cas9 technology and found, consistent with previous reports, no effect on the proliferation of HCC cells or their *in vivo* tumour growth rate. However, knockout did inhibit the migration and invasion of HCC cells. Transcriptome functional enrichment analysis revealed that signaling pathways highly related to cell migration, such as the PI3K-AKT signaling pathway, the ECM-receptor interaction pathway, and the focal adhesion pathway. Mechanistically, CHML upregulates the choline transporter SLC44A3, enhancing choline uptake and phosphatidic acid (PA) production. This metabolic shift activates MAPK and PI3K-AKT signaling cascades, thereby amplifying HCC migratory and invasive capacity. Our findings establish a novel CHML-SLC44A3-choline metabolic axis that drives HCC progression through coordinated transcriptional and metabolic reprogramming, revealing actionable therapeutic targets for clinical translation.

## 2 Materials and methods

### 2.1 Cell culture

The human liver cancer cell line Huh7, procured from the American Type Culture Collection (ATCC, USA), was maintained in Dulbecco’s Modified Eagle Medium (DMEM, Hyclone, USA) enriched with 10% fetal bovine serum (FBS, Gibco, USA). These cultures were kept under standard cell culture conditions of 37°C with 5% CO_2_ in a humidified atmosphere.

### 2.2 Lentiviral transduction of sgRNA

CHML sgRNA was designed online and synthesized into a lentivirus. Huh7 cells were infected with sgRNA at 60% confluence for 48 hours, followed by puromycin selection for 4 days. Cas9 lentivirus was then added for an additional 48 hours of infection. Infected cells were diluted and plated in 96-well plates to form monoclonal cells, and sequenced one month post-infection.

### 2.3 PCR and agarose gel electrophoresis

The PCR mixture consisted of 2x Taq master mix, primers, template, and DEPC water to a final volume of 10 µL. The cycling conditions were: 95°C for 3 min, followed by 35 cycles of 95°C for 15 s, 55°C for 15 s, and 72°C for 30 s, with a final extension at 72°C for 5 min. Products were electrophoresed on agarose gel at 110 V for 35 min, then photographed.

### 2.4 Quantitative real-time PCR

Total RNA was extracted from cells using Trizol, and cDNA was synthesized with a TaKaRa RT-PCR kit following the manufacturer’s instructions. A 10 µL reaction mixture containing SYBR, cDNA, DEPC water, and primers was prepared and analyzed using a Roche 480 LightCycler 480 PCR instrument for amplification and detection. The primer sequences for this study are shown in **Supplementary Table S1**.

### 2.5 CCK-8 assay

Cells (2×10^3/well) were plated in 96-well plates and incubated at 37°C, 5% CO2 for 24, 48, and 72 hours. CCK-8 (10 µL/well) was added at each time point and incubated further. Cell proliferation was assessed by measuring OD at 450 nm.

### 2.6 Colony formation assay

Cells were trypsinized, suspended, and seeded into 6-well plates for 14 days at 37°C, 5% CO2, with media changes every 3 days. After fixation with 4% paraformaldehyde and staining with 0.1% crystal violet, colonies were counted post-washing and drying.

### 2.7 EdU cell proliferation assay

The appropriate number of cells was cultured overnight on a 96-well or 6-well plate, and the 1×EdU (10µM) working solution was added and continued to culture the cells for 2 h. After EdU labeling was completed, the cells were treated according to the kit instructions (C0088S, C0078S, Beyotime). Finally, absorbance was measured directly at 370 nm, and photographs were taken using a fluorescence microscope.

### 2.8 Wound-healing assay

The cells were seeded at a density of 5×10^5^ cells per well. Once the cells reach 80% confluence, add serum-free medium and starve the cells overnight. The monolayers were scraped with a 10 µL sterile tip and photographed for the scratches at 0 h and 24 h under a microscope. Use image analysis software (ImageJ) to measure the width of the scratches and compare the rate of wound healing over time.

### 2.9 Cell invasion and migration assay

Transwell assays with 8 µm pores (14341, LABSELECT) were performed to assess cell invasion and migration, with or without Matrigel (356234, BD Biocoat) coating. Cells (2×10^5/mL) in serum-free medium were seeded in the upper chamber and incubated at 37°C for 24 h. Following fixation with 4% paraformaldehyde and staining with 0.1% crystal violet, non-migrating/invading cells were removed, and the remaining cells were photographed and analyzed.

### 2.10 Animal study

Five-week-old male nude mice were housed in SPF facilities for over 3 days before experiments. They were randomly assigned to groups (n=6) and injected subcutaneously with 100 µL of cell suspension mixed with matrix fluid. Tumor formation, weight, and size were monitored; mice were euthanized by cervical dislocation when tumors reached >1.5 cm in diameter for further analysis.

### 2.11 Western blotting

Protein was extracted by RIPA lysis buffer (NCM Biotech, RI, USA), containing 1% protease inhibitor cocktail (Sigma Aldrich, St. Louis, MO, USA), separated by SDS-PAGE, and transferred to 0.2 μm polyvinylidene difluoride membrane (Perkin Elmer Life Sciences, San Jose, CA, USA). After the transfer is complete, place the PVDF membrane in 5% skim milk to block for 1 h at room temperature and incubate with primary antibodies overnight at 4°C. The following primary antibodies were used: CHML (ER64788, HUABIO, China), p38 (8690S, Cell Signaling Technology), Phospho-p38 (4511S, Cell Signaling Technology), ERK1/2 (3F8B3, Invitrogen), Phospho-pERK1/2 (4370S, Cell Signaling Technology), JNK (ab4821, abcam), and Phospho-JNK (4668S, Cell Signaling Technology). Blots were incubated with HRP-conjugated goat anti-rabbit IgG (H+L) (AS014; ABclonal) and Multi-rAb HRP-goat anti-mouse IgG (H+L) (RGAM001, Proteintech) for 1 h at room temperature. Protein bands were visualized using ImageQuant LAS4000 (GE Healthcare, Chicago, IL, USA) and normalized to GAPDH (AC001, ABclonal).

### 2.12 Transcriptome analysis

The transcriptome sequencing experiment included four parts: RNA extraction and detection, library construction, and PE150 sequencing on the Illumina Novaseq 6000 sequencing platform after a qualified library.

### 2.13 Untargeted metabolomics

Samples were freeze-dried, re-suspended in 1000 µL methanol at -20°C, vortexed for 1 min, centrifuged at 12,000 rpm at 4°C for 10 min, and 450 µL supernatant was vacuum-concentrated. They were then re-suspended in 150 µL 2-chlorophenylalanine solution (4 ppm in 80% methanol, stored at -20°C), filtered through a 0.22 µm membrane, and transferred to vials. QC samples were pooled from 20 µL of each, with the rest analyzed by LC-MS. Metabolites were identified from raw data.

### 2.14 University of alabama at Birmingham cancer data analysis

We obtained the expression and prognosis of CHML from the clinical case data of human liver cancer in TCGA through the UALCAN website (http://ualcan.path.uab.edu/) (11).

### 2.15 Gene expression profiling interactive analysis

GEPIA (http://gepia.cancer-pku.cn/index.html), a Peking University tool, offers RNA-seq data from 9,736 tumors and 8,587 normals for single-gene prognostic analysis (12). We analyzed CHML gene prognostic data, plotting hazard ratios, P-values, and Kaplan–Meier survival curves.

### 2.16 Kaplan-meier analysis

A Kaplan-Meier survival analysis was conducted to investigate the impact of CHML on the overall survival (OS) of patients with liver hepatocellular carcinoma (LIHC) (13).

### 2.17 cBioPortal

cBioPortal (www.cbioportal.org) serves as an extensive online platform for visualizing and analyzing cancer genomics data, drawing from the rich resources of the TCGA database (14). We utilized cBioPortal for genetic alterations’ acquisition, visualization, frequency comparison, and genomic summary.

### 2.18 Statistical analysis

Data analysis was conducted in R, with animal experiments repeated at least twice and other experiments at least three times. Results are mean ± SD. Survival analysis was by Kaplan-Meier and Log-rank test, group comparisons by t-test, and multiple groups by ANOVA. CHML expression’s association with LIHC clinicopathological features was assessed by the chi-square test. Statistical significance was set at *P*<0.05.

## 3 Results

### 3.1 Knockout of CHML significantly inhibited the migration and invasion of HCC cells

**Figure 1:**
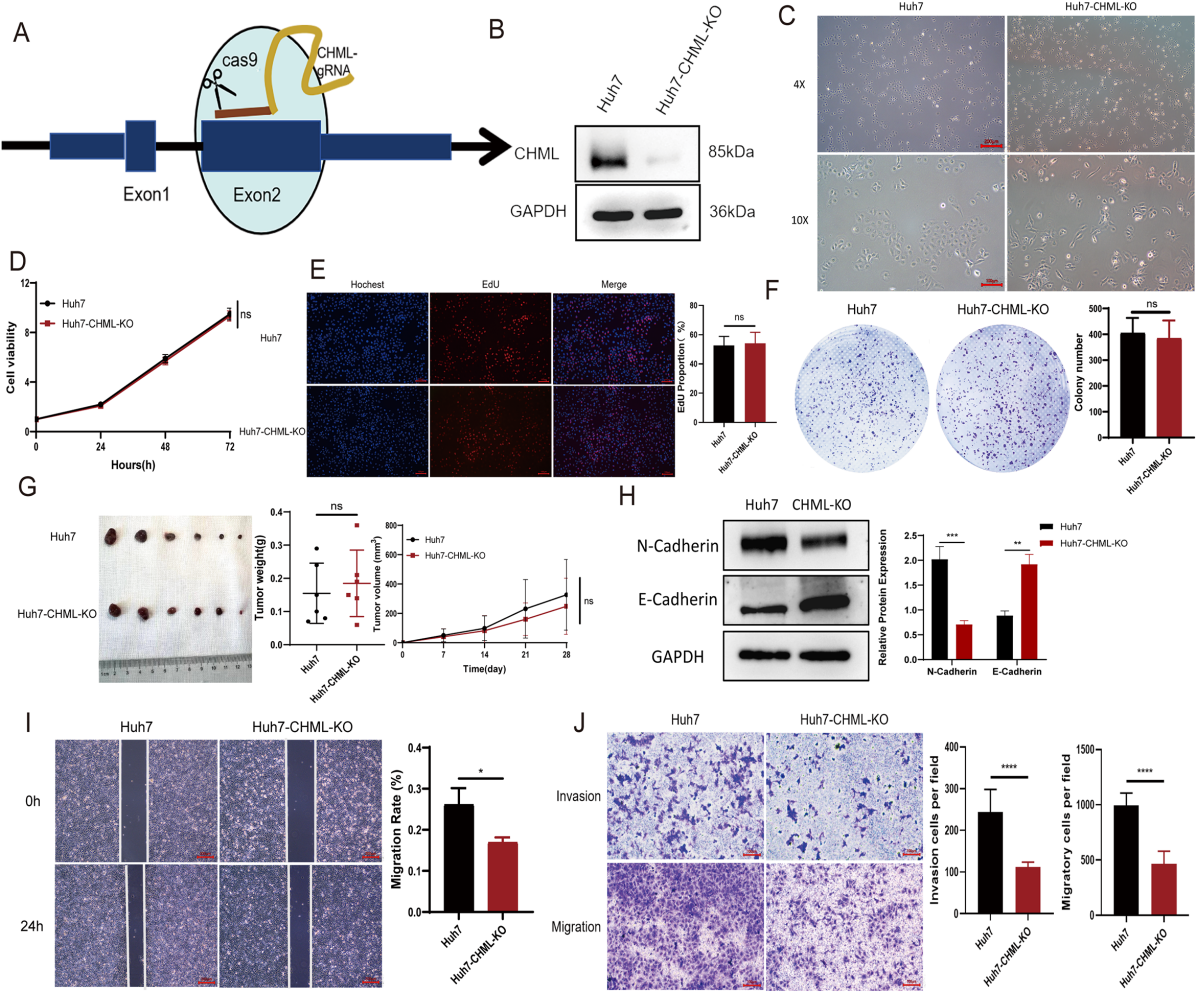
CHML knockout significantly inhibited the migration and invasion of HCC cells. **(A)** The sgRNA targeting exon 2 of the CHML gene was designed. **(B)** Western Blot result showed that CHML was successfully knocked out in Huh7. **(C)** Observation of cellular morphological changes in CHML-knockout Huh7 cells. **(D-G)** CCK-8, EdU, colony formation assay and subcutaneous tumor formation showed that CHML did not affect the proliferation of hepatoma cells (*P* > 0.05). **(H)** Upon CHML knockout, cells exhibited increased E-cadherin expression accompanied by decreased N-cadherin levels. **(I, J)** The invasion and migration ability of Huh7 were weakened after CHML knockout (ns=non-significant, *P< 0.05, **P< 0.01,***P< 0.001, ****P< 0.0001).

To verify CHML’s role in tumor progression, we generated stable CHML-knockout Huh7 liver cancer cells using CRISPR-Cas9 and lentiviral transduction targeting exon 2, with PCR (500 bp band) and sequencing confirming mutations. Western blot analysis revealed significantly reduced CHML protein expression compared to controls (**Figures 1A, B**). Therefore, the above results indicated that CHML was successfully knocked out in Huh7 cells through CRISPR-Cas9 technology, and we established a stable cell line in which CHML expression was knocked out.

CHML knockout induced significant morphological alterations in Huh7 cells, characterized by increased cell size and irregular shape (**Figure 1C**). To explore the effect of CHML on the proliferation of HCC cells, a series of functional experiments were subsequently conducted. The results of CCK-8, EdU-594, and plate colony assays indicated that there were no significant alterations in the activity or proliferative capacity of control cells or the knockout cells (**Figures 1D-F**). In the BALB/c nude mouse subcutaneous tumour model using HCC cells, no significant differences in tumor volume or weight were observed between tumors derived from CHML-knockout Huh7 cells and the Huh7 cells (**Figure 1G**). CHML knockout increased E-cadherin (epithelial marker) and decreased N-cadherin (mesenchymal marker) expression by Western blot (**Figure 1H**). However, the results of *in vitro* wound-healing, cell migration, and transwell analyses demonstrated that the wound healing of HCC cells was inhibited, and the number of cells that passed through the matrix gel to the lower chamber was significantly lower after CHML knockout (**Figures 1I, J**). These results indicated that CHML knockout hindered the migration and invasion abilities of Huh7 cells *in vitro*.

### 3.2 Overexpression of CHML promoted cell migration and metastasis

**Figure 2:**
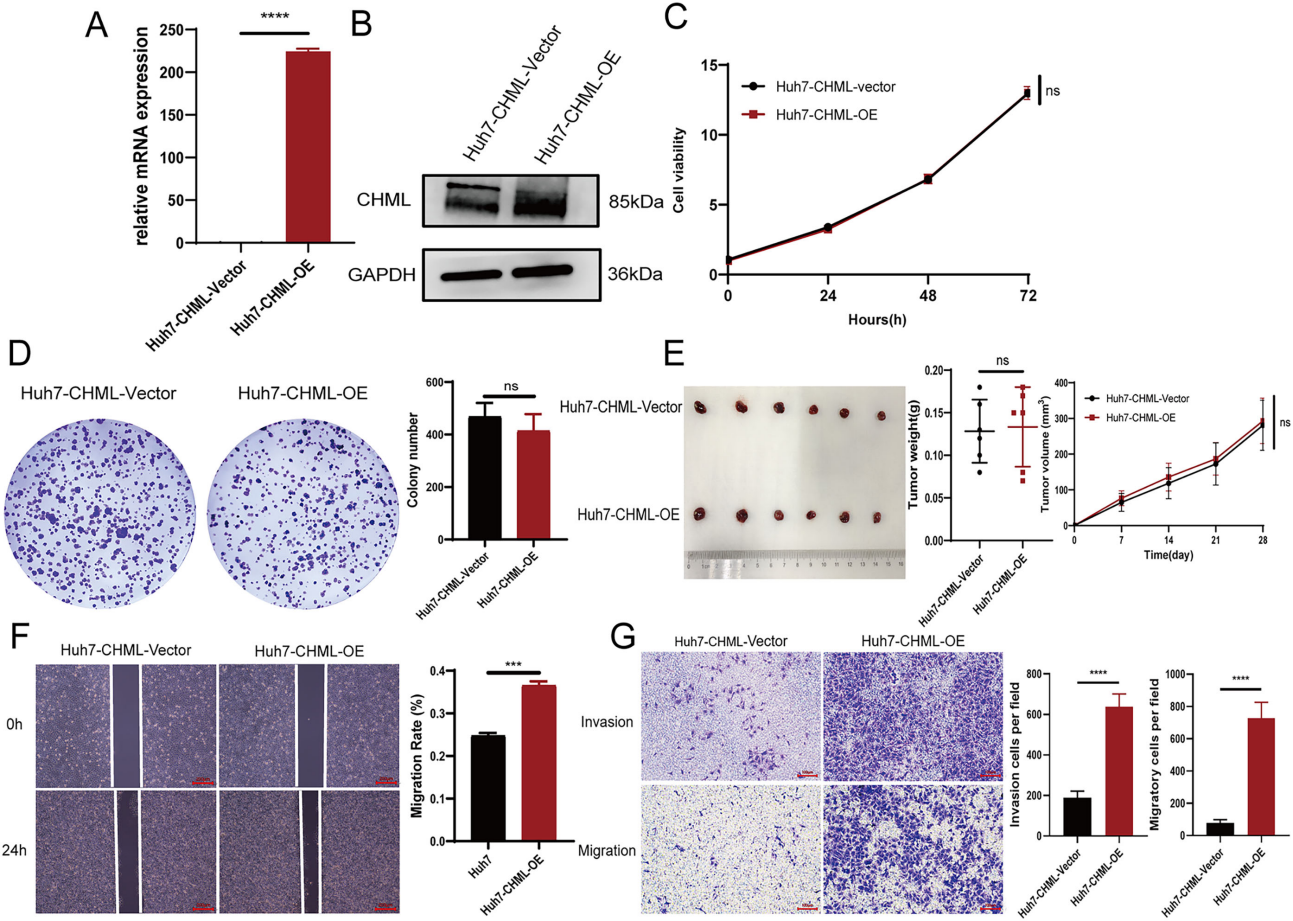
Overexpression of CHML promoted cell migration and metastasis. **(A, B)** qPCR and western bloting results revealed overexpression of CHML at mRNA and protein levels. **(C-E)** CCK-8, colony formation assay and subcutaneous tumor formation showed that CHML did not affect the proliferation of hepatoma cells (*P* > 0.05). **(F, G)** The invasion and migration of Huh7 were enhanced after CHML overexpression (ns=non-significant, ***P< 0.001, ****P< 0.0001).

Furthermore, we utilized lentiviral transfection to overexpress CHML and establish stable Huh7 HCC cells with high CHML. Quantitative PCR and Western Blot revealed a significant increase in CHML expression in these cells (**Figures 2A, B**). The results of the *in vitro* CCK-8 and plate colony experiments revealed that, upon overexpression of CHML in Huh7 cells, there was no difference in the proliferative capacity of liver cancer cells compared with that of control cells (**Figures 2C, D**), suggesting that CHML did not influence the proliferative ability of liver cancer cells. *In vivo* experimental results also demonstrated that CHML overexpression did not affect tumour volume or weight in nude mice relative to control tumours, indicating that CHML might not affect the proliferative ability of HCC cells (**Figure 2E**). The wound-healing assay indicated that compared with that of control Huh7 cells, the migration of Huh7 cells overexpressing CHML was significantly greater (**Figure 2F**). The results of migration experiments revealed that overexpression of CHML significantly altered Huh7 cell migration, increasing chamber penetration approximately 3-fold compared with control cells. Additionally, transwell experiments showed that CHML overexpression significantly increased the invasive capacity of Huh7 cells approximately 7-fold compared with control cells (**Figure 2G**). The above experimental results collectively suggested that CHML significantly promoted the invasion and metastasis of liver cancer cells.

### 3.3 Transcriptome sequencing results suggested that migration-related pathways were significantly enriched after CHML knockout

**Figure 3:**
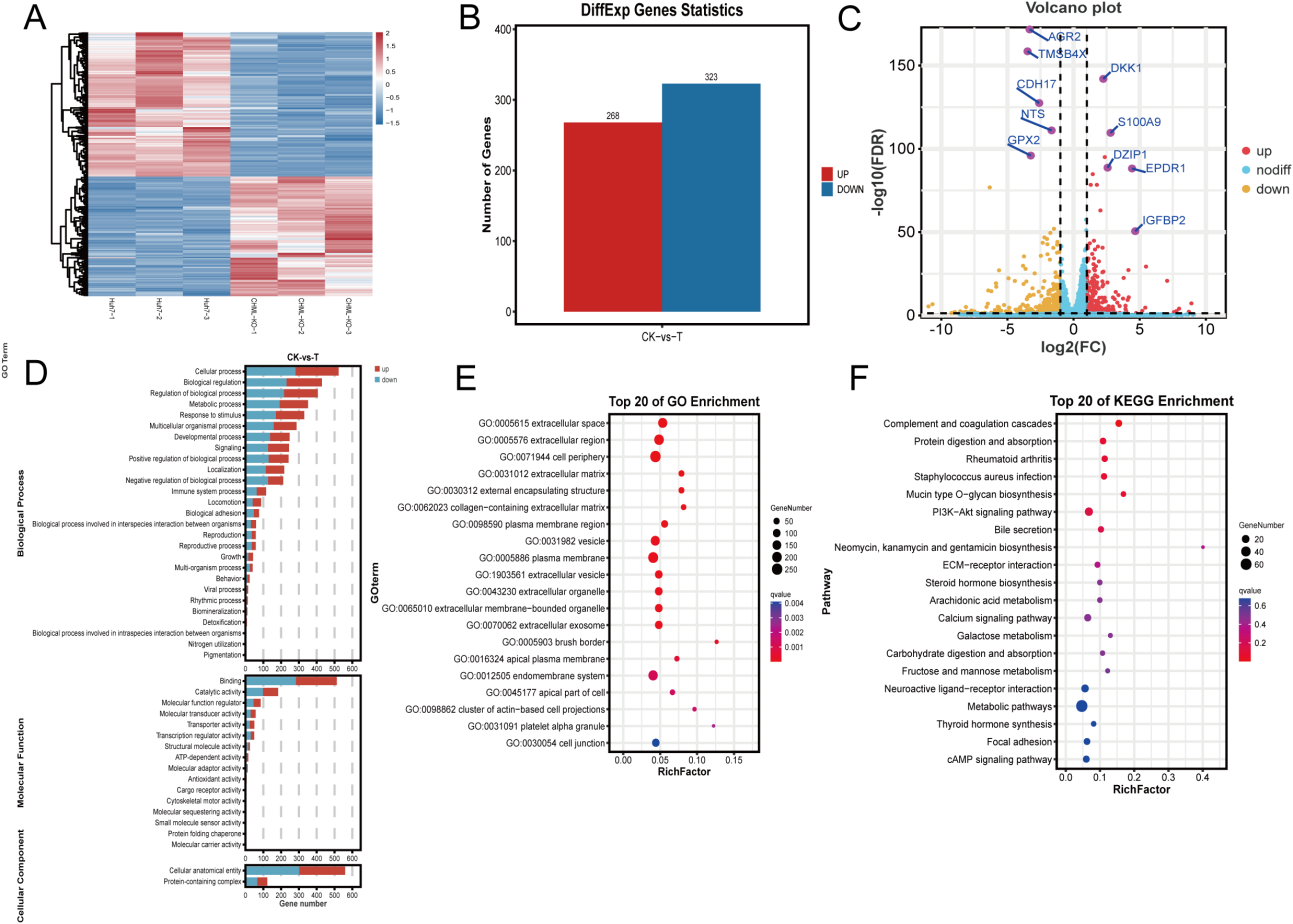
Transcriptome analysis of differentially expressed genes (DEGs) after knocking out CHML. **(A)** Hierarchical clustering of differential gene expression patterns was performed, and a heat map was used to present the clustering results. **(B)** In the Huh7-CHML-KO vs. Huh7 comparison, differential genes with FDR ≤ 0.05 and fold change≥2 were identified, with red and blue indicating up- and down-regulation, respectively. **(C)** The x-axis denoted log2Fold-change, and the y-axis shown -log10FDR. Thresholds were FDR ≤ 0.05 and fold-change≥2. Yellow, red, and blue points signify downregulated, upregulated, and non-significant genes, respectively. **(D, E)** DEGs from CHML knockout were enriched and classified via GO analysis, with bar charts showing DEG counts per GO term. Significant enrichment bubble plots from TOP 20 GO analysis. **(F)** TOP 20 enriched KEGG significant bubble plots were used to describe the proportion and significance of DEGs in each class of Pathway.

To further elucidate the molecular mechanisms underlying CHML-driven migration and metastasis in liver cancer, we conducted transcriptomic sequencing on CHML-knockout Huh7 cells. Cluster analysis confirmed distinct expression patterns between groups (**Figure 3A**). Subsequent differential expression analysis identified DEGs. In total, 591 DEGs with 268 upregulated and 323 downregulated genes were identified (CHML-KO vs. Control dataset; **Figure 3B**). Volcano plots further illustrated the changes in gene expression levels (**Figure 3C**, **Supplementary Figure S1A**). We employed Gene Ontology (GO) analysis and KEGG analysis to annotate the potential signaling pathways involved. Enriched GO terms included extracellular matrix-related annotations critical for cancer cell migration (**Figures 3E, F**, **S1B, C**). KEGG analysis revealed perturbation of PI3K-AKT, ECM-receptor interaction, focal adhesion, and calcium signaling pathways upon CHML knockout. Notably, PI3K-AKT signaling was significantly downregulated (Enrichment fold=2.8, P<0.01) (**Figure 3G**).

### 3.4 CHML modulated the metabolic profiles of HCC

**Figure 4:**
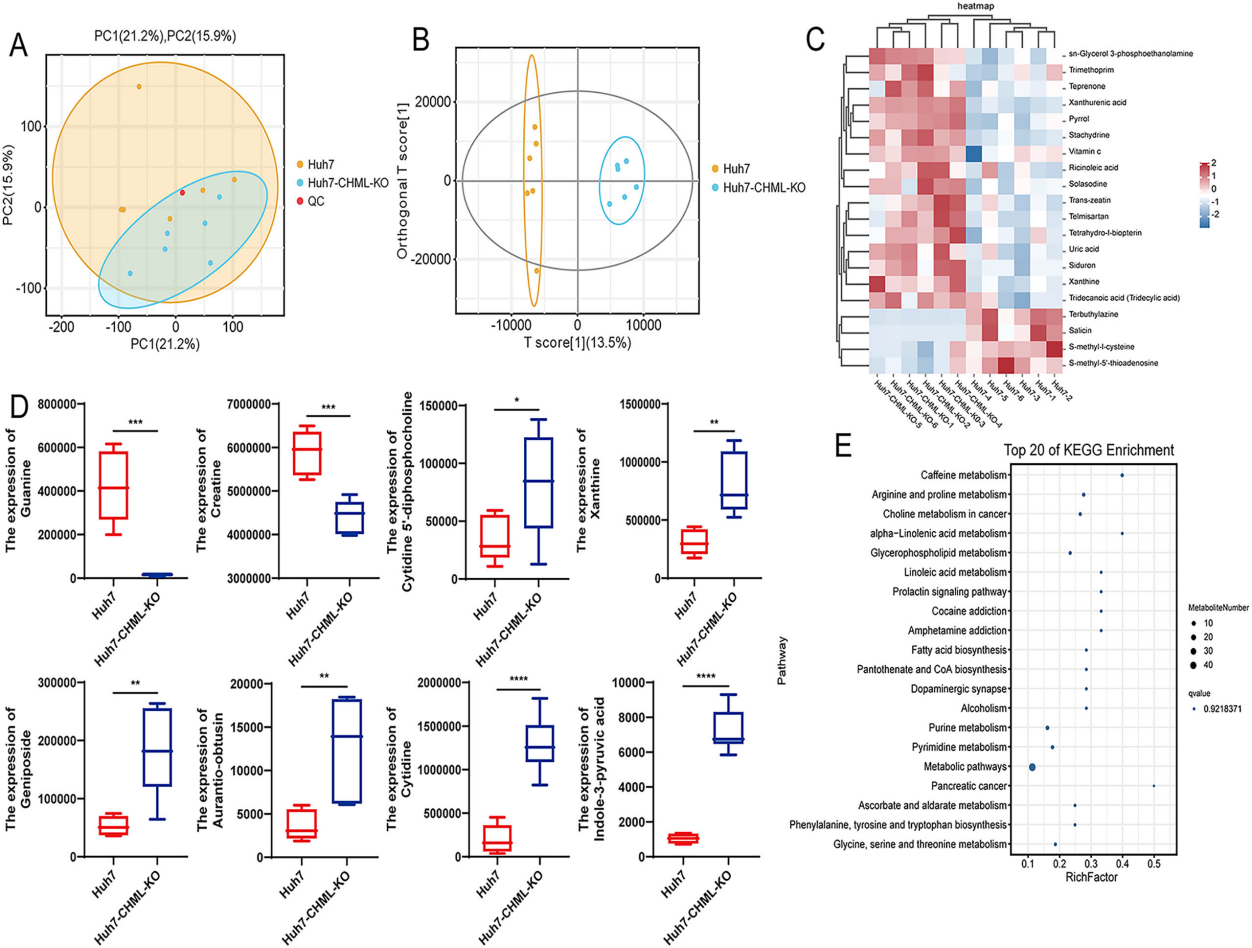
CHML influenced the metabolic signatures in HCC. **(A)** PC1 (21.2%) and PC2 (15.9%) accounted for sample variation in PCA. **(B)** OPLS-DA score plot. **(C)** Metabolite cluster plot displayed samples as rows and genes as columns, with color intensity indicating differential expression levels of metabolites, red for upregulation and blue for downregulation. **(D)** Expression of significant differential metabolites associated with liver cancer in Huh7 and Huh7-CHML-KO cells. ^*^ *P<* 0.05; ^**^ *P<* 0.01; ^***^ *P<* 0.001; ^****^ *P<* 0.0001. **(E)** Top 20 KEGG bubble map delineated differential metabolites’ distribution and significance across metabolic pathways.

Transcriptomic results indicated that after CHML knockout, many metabolism-related signaling pathways, such as steroid hormone biosynthesis, arachidonic acid metabolism, galactose metabolism, and metabolic pathways (**Supplementary Figures S1D–G**), were significantly enriched. Subsequently, we performed untargeted metabolomics using liquid chromatography–mass spectrometry (LC–MS) to investigate how CHML affected the metabolic profile of HCC cells. PCA (**Figure 4A**) and OPLS-DA (**Figure 4B**) revealed that the metabolic profiles of tumours in the control and knockout groups were different. The heatmap visually displayed the different tumour metabolites between the different groups (**Figure 4C**). Furthermore, the boxplot showed the changes in key differentially abundant metabolites.

Additionally, the box plot showed the changes in key metabolites. Compared with control cells, HCC cells with CHML knockout presented lower levels of guanine and creatine but higher levels of cytidine 5’-diphosphocholine, xanthine, geniposide, aurantio-obtusin, and cytidine (**Figure 4D**). The KEGG pathway analysis of metabolic products revealed that pathways such as arginine and proline metabolism, choline metabolism in cancer, and glycerophospholipid metabolism were significantly enriched (**Figure 4E**; **Supplementary Figure S2A**). MetPA revealed that pathways such as D-glutamine and D-glutamate metabolism, purine metabolism, arginine and proline metabolism, and important metabolic pathways were significantly enriched (**Supplementary Figure S2B**); moreover, MSEA revealed that important metabolic pathways such as oxidation of branched chain fatty acids, glycine and serine metabolism, arginine and proline metabolism, and the glucose-alanine cycle were also significantly enriched (**Supplementary Figure S2C**). These data suggested that CHML significantly changed the metabolic characteristics of HCC cells.

### 3.5 Integrated transcriptomics-metabolomics analysis identifies choline metabolism as a central pathway in CHML-driven HCC metastasis

**Figure 5:**
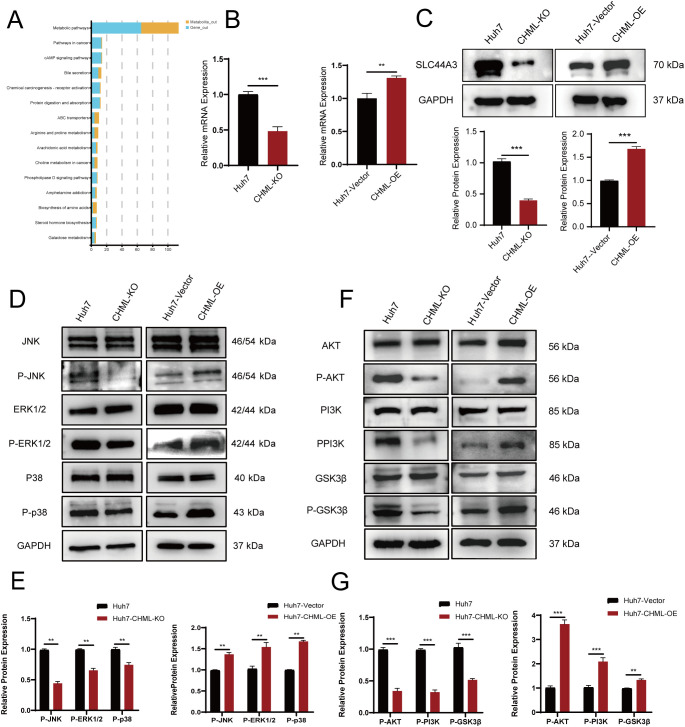
CHML regulates HCC cell migration and invasion through choline metabolism and MAPK/PI3K-AKT signaling pathways. **(A)** Map of enriched signaling pathways from combined transcriptional and metabolic analyses, with blue for genes and yellow for metabolites. **(B, C)** qPCR and Western Blot were used to verify the expression of SLC44A3 in Huh7, Huh7-CHML-KO, Huh7-CHML-Vector and Huh7-CHML-OE cells. ^**^ *P<* 0.01; ^***^ *P<* 0.001. **(D-G)** Expression of MAPK and PI3K-Akt pathway components in Huh7 parental, CHML-KO, vector control, and OE cells.

To further explore the effects of CHML knockout on the occurrence and progression of liver cancer, we conducted a joint analysis of DEGs via transcriptomic sequencing and differentially metabolized compounds based on untargeted metabolomics. This analysis revealed that the choline metabolism pathway in cancer served as a common pathway (**Figure 5A**). To further validate the expression of key genes SLC44A3 in this pathway, real-time PCR and Western Blot were employed to assess its expression levels in cells. The results revealed significant downregulation of SLC44A3 in Huh7-CHML-KO cells and upregulation in Huh7-CHML-OE cells(P<0.05) (**Figures 5B, C**), which was consistent with the findings from the transcriptome sequencing analysis. Subsequently, Western blotting was conducted to examine the expression and phosphorylation levels, pivotal node proteins within the MAPK and PI3K-AKT signaling pathways. Notably, while CHML did not affect the total protein levels of JNK, ERK, p38, PI3K, or AKT, it significantly decreased the expression levels of P-JNK, P-ERK, P-p38, P-PI3K, and P-AKT upon CHML downregulation. Conversely, the overexpression of CHML in Huh7 cells led to increased expression and phosphorylation of these corresponding proteins (**Figures 5D–G**). These findings suggested that by modulating the PA content and activating the MAPK and PI3K-AKT signaling pathways, CHML might play a regulatory role in the migration and invasion of HCC cells.

The O2PLS analysis results identified the top 10 genes and metabolites associated with transcriptomics and metabolomics, respectively. The genes were ADAP1, SLC1A1, MAGEB6, LAMB3, TMPRSS4, ZNF462, ITGB2, HLA-DMB, C1R, and SHD. The metabolites were: limaprost (M760T39_NEG), N-alpha-acetyl-l-lysine (M171T266_POS), 2-arachidonoyl-1-palmitoyl-sn-glycero-3-phosphoethanolamine (M739T148_NEG), N-(phenylacetyl)-l-phenylalanine (M318T369_NEG), NCGC00384956-01 (M249T53_NEG), 4-amino-2-hydroxy-5-[[1-hydroxy-1-(5-oxo-6-bicyclo[4.1.0]hept-3-enyl)propan-2-yl]amino]-5-oxopentanoic acid (M349T472_POS), bracteatin (M320T368_POS), inosine (M267T222_NEG), phosphoserine (M184T383_NEG), and bendiocarb (M167T134_POS) (**Supplementary Figures S2D, E**). A heatmap was used to visually represent the degree of correlation between genes and metabolites with a gradient colour scale. As shown in the figure, the top 25 gene–metabolite relationships based on correlation values were plotted as a heatmap (**Supplementary Figure S2F**). These genes and metabolites could serve as key factors in the treatment of liver cancer.

### 3.6 CHML as a potential interventional target for HCC in clinical settings

**Figure 6:**
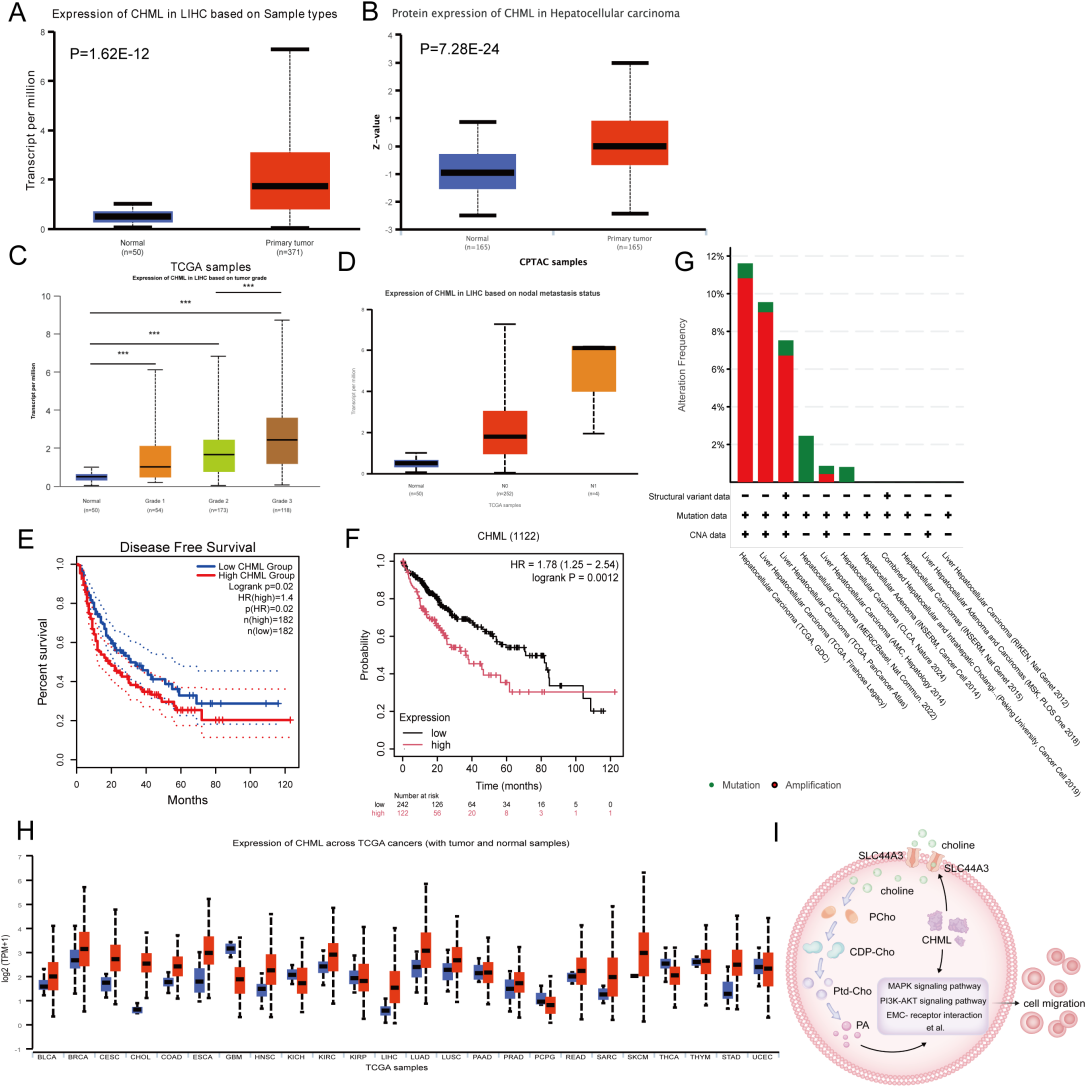
The expression and prognostic situation of CHML in HCC. **(A, B)** Analysis of CHML mRNA and protein expression in HCC patients from TCGA database via UALCAN. (P=1.62E-12, 7.28E-24) **(C)** CHML mRNA levels in HCC subgroups were analyzed across tumor differentiation grades: G1 (well-differentiated), G2 (moderately differentiated), and G3 (poorly differentiated). **(D)** CHML expression levels in HCC were analyzed by nodal metastasis statu. sN0 (no metastasis) and N1 (metastasis) groups were compared using box plots. **(E)** Disease-free survival (DFS) analysis by GEPIA; *P*=0.02 **(F)** Survival analysis by KM; *P*=0.001. **(G)** Proportions of alteration frequencies and genetic types. **(H)** Among differentially expressed data from TCGA database, CHML was more likely to be highly expressed in various cancers, especially in liver cancer. Red indicates high expression; blue indicates low expression. ^***^ *P* < 0.001. **(I)** CHML could influence transcription as well as promoted the metabolic-related choline transporter SLC44A3, affecting the transport of choline into the cytoplasm, which led to an increase of PA, thereby impacting migration-associated signaling pathways such as PI3K-AKT, MAPK, and ECM-receptor interaction et al.

To determine CHML gene expression in liver cancer, we analysed data from the UALCAN database. When CHML expression in HCC cases was compared with that in normal tissues, we observed a marked difference (P=1.62E-12, P=7.28E-24) (**Figures 6A, B**). CHML exhibited the highest mRNA expression levels in poorly differentiated HCC and was further upregulated in HCC with lymph node metastasis (**Figures 6C, D**). These findings demonstrated the potential key role of the CHML gene in the development of HCC.

Using the GEPIA and Kaplan–Meier (KM) methods, we evaluated the prognostic significance of CHML gene expression disparity in the advancement of HCC. Analysis of the DFS curve via GEPIA indicated that elevated CHML expression levels were strongly correlated with a reduced duration of DFS (P=0.02) (**Figure 6E**). KM survival curves revealed a significant correlation between elevated CHML expression and reduced overall survival (P=0.0012) (**Figure 6F**). Genetic variant analysis via OncoPrint in cBioPortal to explore genetic variation in the CHML gene in HCC revealed predominant amplification and mutation of the CHML gene (**Figure 6G**).

We conducted a pancancer analysis of CHML expression via the TCGA database and discovered that CHML was overexpressed in a variety of cancers (**Figure 6H**). Additionally, by analysing the TCGA database, we validated that CHML was positively correlated with several key identified genes, including AGR2, TMSB4X, CDH17, LAMC2, LAMB3, COL9A3, CREB3L1, ADAP1, MAGEB6, TMPRSS4, ZNF462, ITGB2, and HLA-DMB (**Supplementary Figures S3A–M**), and was significantly negatively correlated with the genes C1R, IGFBP2, and SHD (**Supplementary Figure S3N–P**) in clinical samples.

In conclusion, CHML significantly influences the migratory capacity of HCC cells by modulating a variety of migration-related signaling pathways, including MAPK and PI3K-AKT, as well as by orchestrating metabolic reprogramming. Notably, CHML upregulates the expression of SLC44A3, thereby enhancing the cellular uptake of choline and subsequently increasing the levels of the downstream metabolite PA. These alterations ultimately impact cell migration through the modulation of the aforementioned signaling pathways. These findings not only offer novel insights into the role of CHML in HCC but also identify potential therapeutic targets for future treatment strategies (**Figure 6I**).

## 4 Discussion

Liver cancer is typically diagnosed at an advanced stage, with recurrence and metastasis posing the greatest challenges during treatment due to the invasiveness of HCC cells. In this study, we observed that the expression of CHML in liver cancer tissues was elevated compared with that in normal tissues. Through systematic phenotypic assays—including migration, invasion, and proliferation experiments—combined with multi-omics analyses, this study elucidated the key mechanistic role of CHML in HCC. Epithelial-mesenchymal transition (EMT) represents a critical step for tumor cells to acquire invasive and metastatic capabilities. CHML drives EMT, evidenced by E-cadherin downregulation and N-cadherin upregulation, thereby enhancing migration and invasion without significantly affecting proliferation. This aligns with previous findings that CHML primarily facilitates HCC progression through motility regulation rather than proliferative acceleration. Transcriptomic profiling revealed that CHML knockout significantly alters the expression of multiple metastasis-associated genes, including AGR2 (15), TMSB4X (16), CDH17 (17), NTS (18), DKK1 (19), S100A9 (20), EPDR1 (21), and IGFBP2 (22). The dysregulation of these genes likely contributes to CHML-mediated migratory and invasive phenotypes. Integrated metabolomic analysis further identified choline metabolism as a key pathway modulated by CHML, particularly through its effect on PA generation. In summary, these findings deepened our understanding of the role of CHML in HCC.

Although CHML is established in retinal biology and choroidal neovascularization (23), its oncogenic roles are increasingly recognized (24). Emerging evidence indicates that CHML, as a transcriptional target of NRF2, shows significant upregulation in lung adenocarcinoma and is strongly associated with poor prognosis (7). Notably, CHML has been incorporated into a 7-gene prognostic model derived from single-cell RNA sequencing and TCGA data, where it serves as one of the tumor stemness-related genes in triple-negative breast cancer (25). These findings collectively suggest that CHML may function as a pan-cancer prognostic marker with potential clinical relevance. While previous work demonstrated that CHML facilitates HCC metastasis through Rab14-mediated vesicular transport (10), its downstream mechanisms, particularly its role in metabolic reprogramming, remain incompletely elucidated. Metabolic reprogramming is considered a hallmark of malignant tumours; it promotes tumorigenesis by enhancing tumour cell proliferation, metastasis, invasion, and drug resistance. Concurrently, mutations that occur during tumour progression can further enhance metabolic reprogramming, thus facilitating the advancement of tumours (26, 27). In this study, our integrated analysis of transcriptomics and untargeted metabolomics data revealed that the choline metabolism pathway may play a critical role in CHML-regulated migration and invasion processes.

Additionally, our results indicated that CHML could regulate choline metabolism. Abnormal choline metabolism in cancer is associated with its malignant progression. Among them, PA is generated through the cleavage of phosphatidylcholine by phospholipase D2, which is a critical activator of the PI3K-AKT and MAPK survival signaling pathways (28, 29). PA regulates various cellular processes, including membrane transport, actin cytoskeletal remodeling, cell motility, and cell proliferation (30–32). As a crucial signaling molecule, PA can modulate cellular signaling and metabolic states through multiple mechanisms. Although we did not directly measure PA concentration changes or their precise mechanistic roles in cells, existing studies demonstrate that PA activates both MAPK and PI3K-AKT pathways (33, 34). The MAPK cascade has been well-established to promote cell migration and invasion (35, 36). In our study, CHML upregulated key phosphorylated MAPK proteins (P-p38, P-ERK1/2, and P-JNK). Meanwhile, the PI3K-AKT pathway serves as a master regulator of glycogen synthase kinase 3β (GSK-3β) activation. AKT-mediated phosphorylation of GSK3β triggers its ubiquitination and degradation, while GSK3β phosphorylation also enhances Snail1 phosphorylation and nuclear export, thereby activating EMT (37). Thus, the PI3K-AKT-GSK3β axis represents a critical EMT modulator that influences tumor invasiveness—a finding corroborated by our results. Li et al. (38) found that elevated choline content promoted the proliferation of HCC cells by reprogramming the Krüppel-like Factor 5 (KLF5)-dominated core transcriptional regulatory circuit (CRC). Lau et al. (39) significantly decreased the development of nonalcoholic fatty liver disease-related hepatocellular carcinoma (NAFLD-HCC) in DEN-treated mice fed a choline-deficient high-fat diet supplemented with Lactobacillus acidophilus. These results further illustrate the important role of choline metabolism in the development of liver cancer.

However, our study has several limitations. First, we were unable to directly validate PA concentration changes or their subcellular mechanistic roles. Second, *in vivo* validation of CHML’s impact on metastasis was limited by practical constraints. These gaps warrant future investigation to fully delineate CHML-mediated metabolic reprogramming in HCC.

## Data Availability

The original contributions presented in the study are publicly available. This data can be found here: [https://www.ncbi.nlm.nih.gov/sra/PRJNA1297392] [https://www.ebi.ac.uk/metabolights/MTBLS12789].
