# Supplementary material for: CHML regulates migration and invasion in hepatocellular carcinoma via transcriptional and metabolic reprogramming
Source: Front Oncol. 2025 Aug 6;15:1575809. doi: 10.3389/fonc.2025.1575809 (PMC12364631; doi:10.3389/fonc.2025.1575809)
Supplement: Supplementary file 1 [file DataSheet1.pdf]

Supplementary Information

CHML regulates migration and invasion in hepatocellular carcinoma via transcriptional and metabolic reprogramming

Huanqian Cao1†, Siyu Wang1†, Li Zhang2,3†, Heying Xie1, Yiqiong Liu1, Ruijiao Kong1, Yin Jia1, Ling Lu1, Junfeng Jiang4,5*, Shanrong Liu1*

[1]Department of Laboratory & Diagnosis, Changhai Hospital, Navy Medical University, Changhai Road 168, Shanghai, 200433, China;

[2]Department of Pathology, Faculty of Medical Imaging, Naval Medical University, Shanghai, 200433, China;

[3]Department of Pathogen Biology, Naval Medical University, Shanghai 200433, China;

[4]Histology and Embryology Department, Naval Medical University, Shanghai, 200433, China;

[5]School of Gongli Hospital Medical Technology, University of Shanghai for Science and Technology, Shanghai 200093, China;

[*] Correspondence:
Shanrong Liu and Junfeng Jiang

E-mail: liushanrong01@126.com and jeffrey99@qq.com

Table S1 Primers of qRT-PCR analysis.

	Forward Primers	Reverse Primers
β-actin	CATGTACGTTGCTATCCAGGC	CTCCTTAATGTCACGCACGAT
CHML	CCTTCAGTGTCTCGGACGGTTT	ATGACGAAGACAATAGATTCCACC
LAMC2	TACAGAGCTGGAAGGCAGGATG	GTTCTCTTGGCTCCTCACCTTG
LAMB3	GTCACAGAGCAGGAGGTGGCT	GCTTCTGTCAAGACTCTCCAGG
COL9A3	GAGTCCTCCCTGAAGGCGCTA	CTGCTCGCCTTTGTAGCCAGTG
CREB3L1	GCCTTGTGCTTTGTTCTGGTGC	CCGTCATCGTAGAATAGGAGGC
FGF18	ACGATGTGAGCCGTAAGCAGCT	ACCGAAGGTGTCTGTCTCCACT
RELN	GTCTACCTTCCACTCTCCACCA	GTCCAGCATCACAAATCCCTCG
ERBB4	GGAGTATGTCCACGAGCACAAG	CGAGTCGTCTTTCTTCCAGGTAC
PIP5K1B	CGTCAATGAGCACTATCCACACG	TCAGCGGTCAAGTAGATGGTCC
JMJD7-PLA2G4B	GGATTGCGTCTCCTACATCACC	CCAGCTTGTTCTTGGTCACCTG
SLC44A3	TCTCTGTGGTGAGGATTCCGAG	CAGTAGCAGCATCGGAACAGGT

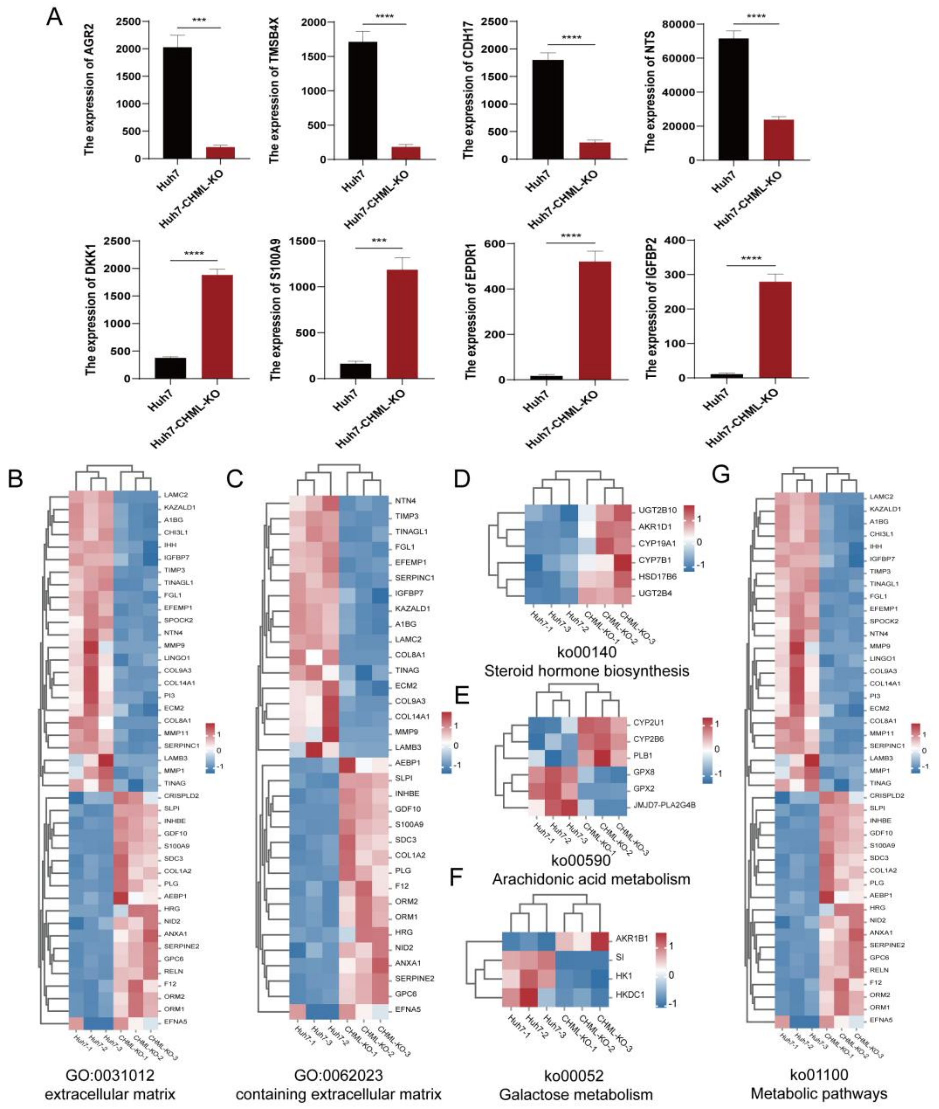

Fig. S1. Enriched GO and KEGG pathways and associated genes

(A) Expression of significantly different genes in the volcano plot of transcriptome sequencing results. $^{*}P < 0.05$; $^{**}P < 0.01$; $^{***}P < 0.001$; $^{****}P < 0.0001$. (B-C) Cluster map of GO enrichment pathways for Extracellular Matrix (ECM) and collagen-containing extracellular matrix. Each row represented a gene, each column represented a sample, and changes in color intensity of red and blue represented changes in gene expression abundance. (D-G) Cluster map of KEGG signaling pathways for steroid hormone biosynthesis, the arachidonic acid metabolism, galactose metabolism, and metabolic pathway. Each row represented a gene, each column represented a sample, and changes in color intensity of red and blue represented changes in gene expression abundance.

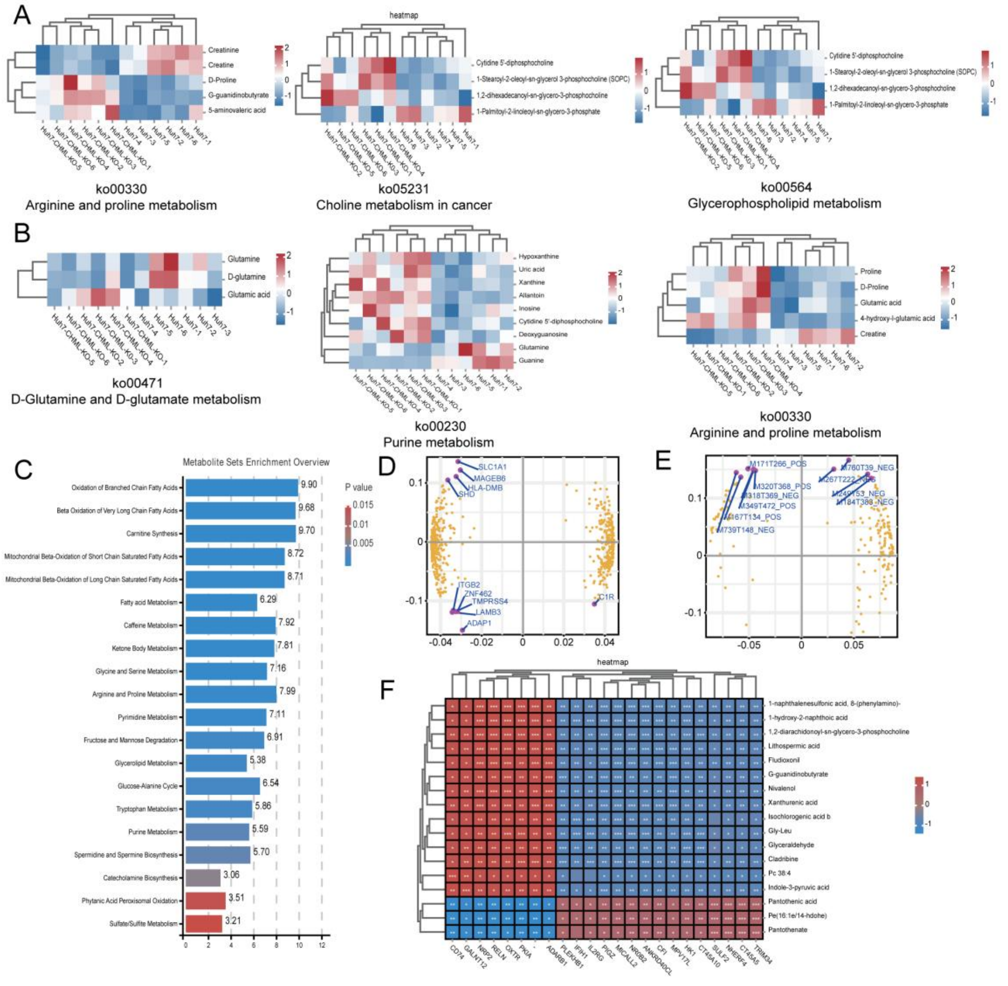

Fig. S2. Enriched pathways and associated metabolites.

(A) KEGG important pathways of non-targeted metabolomics were significantly enriched. (B) The important metabolic pathway of MetPA was significantly enriched. (C) Select the top 20 enriched signaling pathways based on *P*-value. The horizontal axis represented the degree of enrichment, and the vertical axis represented the pathway names. (D) Identify the top 10 key genes that had a significant impact on the metabolome data from the transcriptome data. (E) The top 10 key metabolites with great influence on the transcriptome data were screened from the metabolome data. (F) A heat map of the TOP 25 gene-metabolite relationship pairs was drawn by Pearson analysis of the correlation size between genes and metabolites. $^{*}P < 0.05$; $^{**}P < 0.01$; $^{***}P < 0.001$; $^{****}P < 0.0001$.

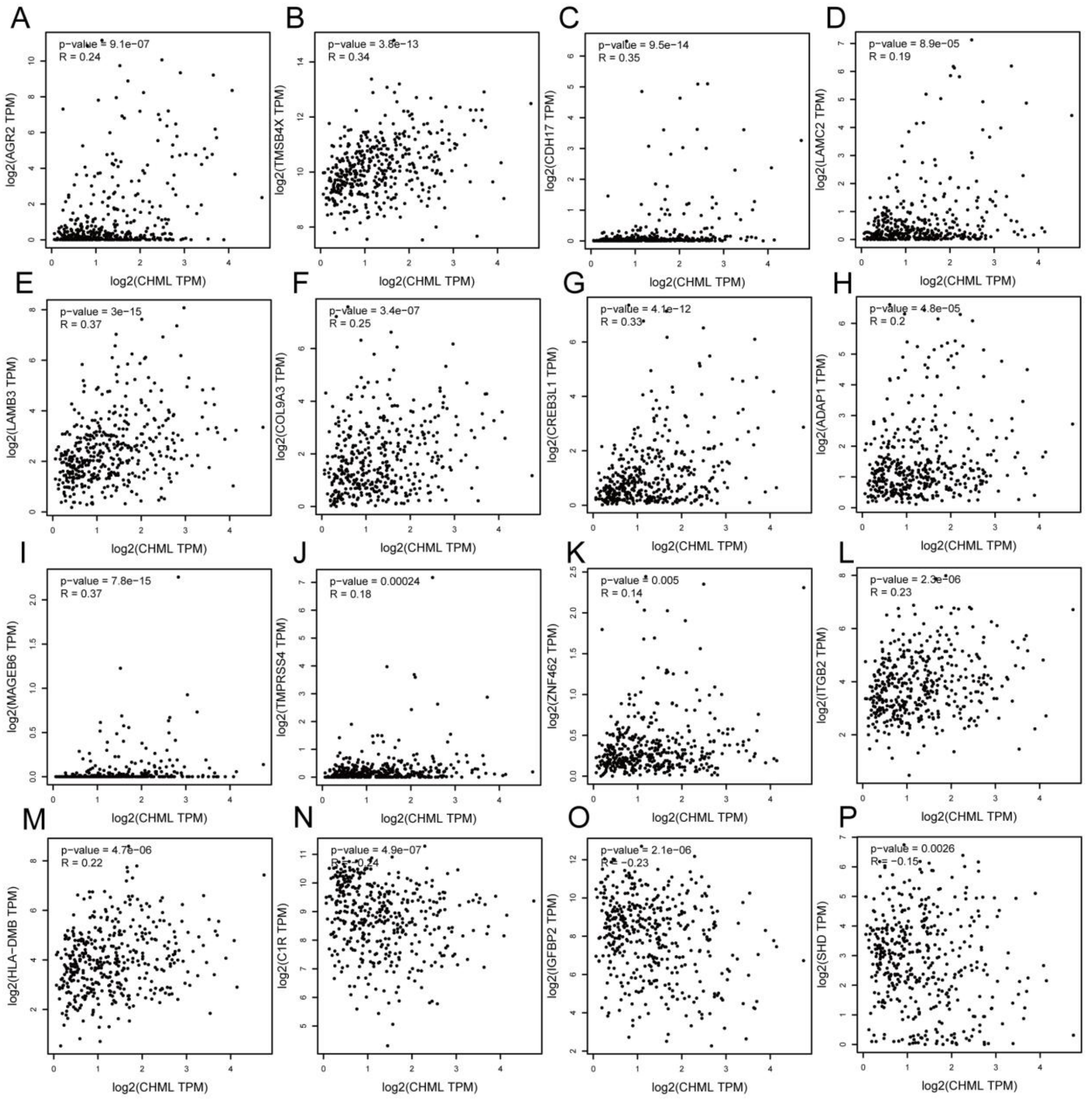

Fig. S3. The correlation between CHML and genes associated with HCC.

Analysis of the Spearman correlation coefficient between CHML and differentially expressed genes in signaling pathways enriched by transcriptome sequencing in HCC patients from the TCGA database via GEPIA. (A) AGR2 (R=0.24; *P*=9.1E-07) (B) TMSB4X (R=0.34; *P*=3.8E-13) (C) CDH17 (R =0.35; *P*=9.5E-14) (D) LAMC2 (R=0.19; *P*=8.9E-05) (E) LAMB3 (R =0.37; *P*=3E-15) (F) COL9A3 (R =0.25; *P*=3.4E-07) (G) CREB3L1 (R=0.33; *P*=4.1E-12) (H) ADAP1 (R =0.2; *P*=4.8E-05) (I) MAGEB6 (R =0.37; *P*=7.8E-15) (J) TMPRSS4 (R =0.18; *P*=0.00024) (K)ZNF462 (R=0.14; *P*= 0.005) (L) ITGB2 (R=0.23; *P*=2.3E-06) (M) HLA-DMB (R=0.22; *P*=4.7E-06) (N) C1R (R=-0.24; *P*=4.9E-07) (O) IGFBP2 (R=-0.23; *P*=2.1E-06) (P) SHD (R=-0.15; *P*=0.0026).
